# Extracts of Zuo Jin Wan, a traditional Chinese medicine, phenocopies 5-HTR1D antagonist in attenuating Wnt/β-catenin signaling in colorectal cancer cells

**DOI:** 10.1186/s12906-017-2006-7

**Published:** 2017-11-28

**Authors:** Jielu Pan, Yangxian Xu, Haiyan Song, Xiqiu Zhou, Zemin Yao, Guang Ji

**Affiliations:** 1 Institute of Digestive Diseases, Longhua Hospital, Shanghai University of Traditional Chinese Medicine, Shanghai, 200032 China; 2 Department of General Surgery, Longhua Hospital, Shanghai University of Traditional Chinese Medicine, Shanghai, 200032 China; 3 Department of Biochemistry, Microbiology and Immunology, Ottawa Institute of Systems Biology, University of Ottawa, Ottawa, ON K1H 8M5 Canada; 4 E-Institute of Shanghai Municipal Education Commission, Shanghai University of Traditional Chinese Medicine, Shanghai, 201203 China

**Keywords:** 5-HTR1D, Colorectal cancer, GR127935, Zuo Jin Wan, Wnt/β-catenin pathway

## Abstract

**Background:**

In vitro and in vivo studies have shown that Zuo Jin Wan (ZJW), a herbal formula of traditional Chinese medicine (TCM), possessed anticancer properties. However, the underlying mechanism for the action of ZJW remains unclear. Various subtypes of 5-Hydroxytryptamine receptor (5-HTR) have been shown to play a role in carcinogenesis and cancer metastasis. 5-HTR1D, among the subtypes, is highly expressed in colorectal cancer (CRC) cell lines and tissues. The present study aimed at investigating effect of ZJW extracts on the biological function of CRC cells, the expression of 5-HTR1D, and molecules of Wnt/β-catenin signaling pathway.

**Methods:**

In this study, the effect of ZJW extracts on 5-HTR1D expression and Wnt/β-catenin signaling pathway were investigated and contrasted with GR127935 (GR), a known 5-HTR1D antagonist, using the CRC cell line SW403. The cells were respectively treated with GR127935 and different doses of ZJW extracts. Proliferation, apoptosis, migration, and invasion of SW403 cells were compared between ZJW and GR127935 treatments. The expression of 5-HTR1D and signaling molecules involved in the canonic Wnt/β-catenin pathway were determined by Western blot analysis.

**Results:**

After ZJW extracts treatment and GR127935 treatment, G1 arrest in cell cycle of SW403 was increased. Cell apoptosis was pronounced, and cell migration and invasion were suppressed. SW403 cells showed a dose-dependently decreased expression of 5-HTR1D, meanwhile, β-catenin level was significantly decreased in nucleus of cells cultured with GR127935. Treatment of ZJW extracts dose-dependently resulted in decreased 5-HTR1D and a concomitant reduction in the Wnt/β-catenin signal transduction, an effect indistinguishable from GR127935 treatment.

**Conclusion:**

The anticancer activity of ZJW extracts may be partially achieved through attenuation of the 5-HTR1D-Wnt/β-catenin signaling pathway.

## Background

Colorectal cancer (CRC) is one of the most common malignancies worldwide. World Health Organization (WHO) reported that CRC ranked the third place in male and second place in female of cancer morbidity, and the fourth of the mortality rates [1]. Current treatments for CRC include surgery, radiation and chemotherapy, but the prognosis is poor [2, 3].

Zuo Jin Wan (ZJW), a herbal formula of traditional Chinese medicine (TCM), has been used in treating gastrointestinal diseases and liver diseases in China for a long history [4, 5]. It is composed of *Rhizoma Coptidis* (Huanglian in China) and *Evodia Rutaecarpa* (Wuzhuyu in China) in ratio of 6 to 1. Berberine and evodiamine are two key components of ZJW extracts that possess anti-tumorigenic activity [6]. In vitro and in vivo experiments have shown that berberine and evodiamine can arrest cell cycle, reduce expressions of some oncogenes, and inhibit tumor metastasis [7, 8]. Animal experiments with ZJW also show its antitumor effect in tumors including CRC [9, 10]. ZJW extracts can inhibit the growth of multi-drug resistant CRC cell lines, increase the sensitivity of chemotherapy, inhibit the tumor growth of xenograft mice, and reduce the P-gp protein expression and reverse drug resistance of CRC cells [11]. However, to date, the mechanism whereby ZJW extracts exert the anti-tumor effect is unclear.

Serotonin, also known as 5-hydroxytryptamine (5-HT), is a biogenic amine produced by enterochromaffin cells (EC) of the gastrointestinal tract [12]. It is a versatile neuro-transmitter, with a role of signal-transduction and maintenance of cell growth. 5-HT exerts its effects through the membrane-bound 5-HT receptors (5-HTRs) consisting of fourteen members [13, 14]. Over the past decades, accumulating preclinical and clinical evidences have pointed out that 5-HT not only plays a role in physiological cell mitosis, but also has a close correlation with cancers [14]. Certain subtypes of 5-HTRs have been reported in the process of different types of cancers, including prostate [15], colon [16], liver [17] and gallbladder cancer cells [18], breast cancer [19], and bladder cancer [20]. 5-HT and 5-HTRs may be a potential factor in the tumorigenesis and tumor progression. It has been found that the agonists of 5-HTR3, 5-HTR4 and 5-HTR1B can promote the proliferation of CRC cells [21], whereas the antagonists of 5-HTR1B can induce apoptosis [22].

Several studies have suggested a potential link between 5-HTRs and CRC. For instance, Xu et al. [23] have reported that a decreased risk of CRC was associated with the use of high daily doses of selective serotonin-reuptake inhibitors (SSRI) 0–5 years before a diagnosis of CRC (incidence-rate ratio 0.70 [95% CI 0·50–0·96]). In another study, it has been shown that a decrease in 5-HTR1A, 5-HTR2C, and serotonin reuptake transporter (SERT) in Caco-2 cells was associated with sulforaphane treatment in a dose-dependent manner [24]. It has been suggested that activation of 5-HTRs, followed by initiation of cyclic AMP signaling, might be crucial events in colon cancer progression [24]. Thus, 5-HTR-mediated signaling pathway might potentially be a novel therapeutic target for colon cancer therapy.

The Wnt/β-catenin pathway (or canonical Wnt pathway) plays an important role in the regulation of cellular growth, apoptosis, cell adhesion, and metabolism [25, 26]. Aberrations of the Wnt/β-catenin pathway cause various diseases including cancer, and mutations in this signaling are frequently observed in cancer [27, 28]. Therefore, the Wnt/β-catenin pathway has been recently considered as the one mostly relevant to cancer [29–31]. Among all human cancer types, it is only CRC for which there is unquestionable evidence that deregulated Wnt signaling drives tumorigenesis [32]. In the canonical Wnt signaling pathway, the central player is β-catenin, a transcription cofactor that, together with T cell factor/lymphoid enhancer factor (TCF/LEF), controls expression of various target genes [33]. The level of β-catenin is negatively regulated by a scaffolding complex, consisting of Axin, adenomatous polyposis coli (APC) and glycogen synthase kinase 3β (GSK3β), which targets β-catenin for degradation through the ubiquitination/proteasome dependent pathway. Wnt binds to Frizzled receptor and inactivates the β-catenin destructive complex via the activation of the dishevelled (Dvl) protein [31].

Recently, higher expression of 5-HTR1D has been observed in human CRC tissues [34]. Experiments with a CRC cell line LoVo have shown that treatment of 5-HTR1D antagonist GR127935 resulted in decreased expression of 5-HTR1D and decreased ability of CRC cell metastasis, which might be mediated by attenuating Wnt/β-catenin signaling [34]. We postulated that ZJW extracts might exert an anti-tumorigenic effect similar to that of a 5-HTR1D antagonist. In this study, we determined the effect of ZJW extracts on the biological function of CRC cells, the expression of 5-HTR1D, and expression of molecules of Wnt/β-catenin signaling pathway.

## Methods

### Cell culture

Human colorectal cancer cell line, SW403, was obtained from College of Chinese Medicine, Shanghai University of Traditional Chinese Medicine (Shanghai, China). Cells were incubated in RPMI 1640 (Biowest, Nuaillé, France) supplemented with 10% (*v*/v) fetal bovine serum (FBS) (Gibco, Auckland, New Zealand), 2 mmol/L L-glutamine, 1 mmol/L sodium pyruvate, 100 units of penicillin, and 100 mg/mL streptomycin (Biowest). The cells were cultivated in an atmosphere of 5% CO_2_ incubator (Thermo scientific Heraeus, Germany) at 37 °C.

### Preparation of medicines for treatment

The 5-HTR1D antagonist GR127935 (GR) (TOCRIS, Ellisville, USA) was dissolved in phosphate buffer saline (PBS) and then diluted into different concentrations. ZJW was prepared using a formula of Chinese herb *Rhizoma Coptidis* (60 g) and *Evodia rutaecarpa* (10 g), at a ratio of 6:1. All the herbs were purchased from Longhua Hospital herbal pharmacy department. Briefly, the mixture (70 g) was extracted twice for 1 h each time by refluxing in ethanol (1: 8, *v*/v). The filtrates were concentrated and dried in vacuum at 60 °C. Its preparations were standardized, regulated, and quality controlled according to the guidelines defined by China Food and Drug Administration (CFDA). High-performance liquid chromatography (HPLC) was used to identify the components of ZJW extract, and confirm the final concentration of ZJW extract to ensure the quality and stability. Detailed procedures were followed with the published protocol [6]. ZJW was dissolved in PBS and diluted into three different concentrations, 25, 50 and 100 μg/mL, corresponding to low, medium and high dose of ZJW (ZJW-L, ZJW-M and ZJW-H).

### Western blot analysis

Cells of different treatment from three separate experiments were lysed with Radio-Immunoprecipitation Assay (RIPA) lysis buffer (Beyotime, Hangzhou, China) to extract protein. The protein in nucleus was extracted with a Kit (Beyotime, Hangzhou, China). Protein concentration was determined using a BCA protein assay kit (CoWin Bioscience, Beijing, China).The cell lysates were dissolved in a loading buffer containing 2% SDS and incubated at 95 °C for 5 min). Samples were separated by sodium dodecyl sulfate–polyacrylamide by gel electrophoresis (SDS–PAGE), subsequently transferred to polyvinylidene fluoride (PVDF) membrane, blocked with 5% non-fat dry milk in Tris-buffered saline-Tween-20 (TBST) for 1 h, and then probed with primary antibodies at 4 °C overnight. The following primary antibodies were used: 5-HTR1D (#ab13895) (Abcam, Cambridge, MA, USA), Axin1(C76H11, #2087), Dvl2 (30D2, #3224), Dvl3 (#3218), GSK-3β (27C10, #9315), phospho-GSK-3β (Ser9) (D3A4, #9322), LEF1 (C12A5, #2230), TCF4 (C48H11, #2569), cyclin D1 (92G2, #2978), c-Myc (D84C12, #5605), β-catenin ((D10A8, #8480), Bcl-2 (50E3, #2870), phospho-Bcl-2 (Ser70) (5H2, #2827), phospho-Bcl-2 (Thr56) (#2875), Bcl-xL (54H6, #2764), Mcl-1 (D35A5, #5453) (Cell Signaling Technology, Danvers, MA, USA), CDK4 (#11026–1), MMP-2 (#10373–2), MMP-7 (#10374–2), CXCR4 (#11026–1), E-cadherin (#20874–1), ICAM-1 (#10020–1) (Proteintech, Wuhan, China), β-actin (#R1102) (Hua An, Hangzhou, China) and Histone 2A (#21260) (SignalWay Antibody, College Park, MD, USA). The membrane was washed with TBST and then incubated with goat anti-rabbit or anti-mouse peroxidase-conjugated secondary antibody (Cell Signaling Technology) for 1 h. Immmunoreactive bands were visualized with enhanced chemiluminescence HRP substrate (Millipore, Billerica, MA, USA) and acquired by GBOX Chemi XT4 System (Syngene, Cambridge, UK). GeneTools software (Syngene) was used to quantify the optical density of the bands. Beta-actin was determined as a loading control for whole lysate, and Histone 2A was used as the internal control for nuclear protein.

### Cell viability assay

CCK-8 assay was performed to measure the cytotoxicity of ZJW on SW403 seeded in 96-well plates with 1 × 10^4^ cells/well. After 24 h, cells were washed with PBS gently and then exposed to either 10% FBS alone or ZJW at different concentrations (25, 50, 100, 200, 400 and 800 μg/mL). After 24 h, previous medium was replaced with 100 μl of fresh medium and 10 μl of CCK-8 reagent (Dojindo, Kumamoto, Japan), followed by 4 h of incubation. Then the optical density (OD) of the cultures was determined by the microplate reader (BioTek, Winooski, USA) at 450 nm. All experiments were done with 6 replicates per experiment and repeated at least 3 times. IC10 was defined as 10% of maximal inhibitory concentration, representing the concentration of an inhibition rate that is required for 10% of cell viability. We set IC10 as a non-cytotoxic concentration of ZJW.

### Cell cycle analysis

SW403 cells were cultured with ZJW-M and ZJW-H and GR (5 μmol/L) in 6-well plates with 2× 10^5^ cells/well for 24 h. Then cellular total DNA contents of the cells were assessed using flow cytometry following propidium iodide (PI) staining. After 24 h, the cells were collected by trypsinization, centrifuge at 1000 rpm for 5 min, washed with cold PBS twice, and resuspended gently with 70% ethanol at 4 °C for 2 h. Fixed cells were centrifuged and washed by PBS twice. 400 μL propidium iodide (PI) was added to the cells solution, gently shaked, and stained for 30 min at 4 °C avoiding the light. Cells were conducted using FACSCalibur Flow Cytometry (BD Biosciences, USA). PI fluorescence was linearly amplified and both the area and width of the fluorescence pulse were measured. 1 × 10^5^ cells were acquired, and the percentages of G1, S and G2 phases were determined using the DNA analysis. Fluorescence measurements were taken at excitation wavelength of 488 nm. Three separate experiments were performed in SW403 cells for cycle analysis. The results were analyzed with CellQuest Pro software.

### Cell apoptosis analysis

SW403 cells were incubated with ZJW-L, ZJW-M, and ZJW-H and GR (5 μmol/L) in 6-well plates with 2× 10^5^ cells/well in triplicates for 24 h. The cells were collected by trypsinization, centrifuge at 1500 rpm for 5 min, washed with cold PBS twice. Then the cells were resuspended in 500 μL Binding Buffer and mixed with 5 μL Annexin V-FITC (KeyGEN Biotech, Nanjing, China) and 5 μL PI, gently shaked. After 10 min incubation at room temperature in the dark, the cell apoptosis were analyzed using FACSCalibur Flow Cytometry (BD Biosciences, USA). Apoptotic cells were represented by high FITC Annexin V and low PI fluorescence signals (Ex 488 nm/Em 530 nm). Three separate experiments were performed for cell apoptosis. The results were analyzed with CellQuest Pro software.

### Cell migration and invasion assays

Cell migration was assayed in transwell chambers (Corning, Wujiang, China). SW403 cells (3 × 10^5^/ mL) suspended in 1640 medium (100 μL, serum free) or medium with ZJW-M, ZJW-H, GR (5 μmol/L), were placed in the upper transwell chamber, and incubated for 18 h. The cells on the upper surface of the filter were completely wiped away with a cotton swab. The filter was then fixed in methanol, stained with 0.1% crystal violet (Sigma, St. Louis, MO, USA), and the cells migrated to the underside of the filter were imaged using a microscope (Olympus, Tokyo, Janpan) at a magnification of 200× (5 fields/filter). The cell number was counted through ImageJ 1.47 (NIH). Experimental procedures of in vitro invasion assay are the same as the migration assay described above except that the filter was previously coated with a layer of Matrigel (Becton-Dickinson) before cell seeding.

### Statistical analysis

Values are expressed as means and standard deviation (SD) and analyzed using one-way ANOVA followed by LSD test for comparisons between groups. Statistical analysis was conducted by SPSS 18.0 and GraphPad Prism 5.0. It was considered significant difference when *P* < 0.05.

## Results

### 5-HTR1D expression after the ZJW extracts treatment in SW403 cells

**Fig. 1:**
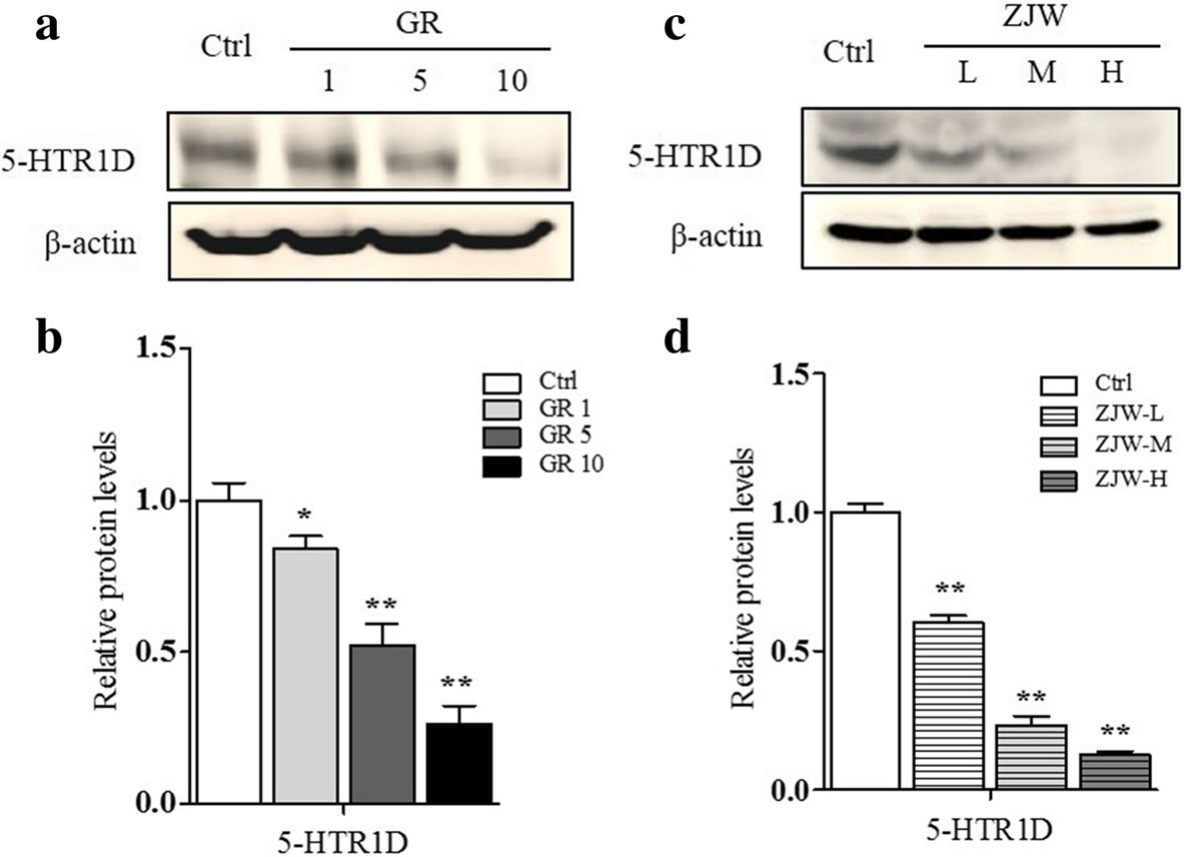
Effect of the ZJW extracts treatment on 5-HTR1D expression in SW403 cell. SW403 cell was stimulated with GR (1, 5, 10 μmol/L) and ZJW at low, medium, and high dose, respectively. Cells were cultivated for 24 h. Protein expression in cells with different stimulation was determined by Western blot. Results were normalized to β-actin expression. **a** Representative western blots of 5-HTR1D protein in GR-treated SW403 cells. **b** The corresponding semi-quantification data of (**a**). **c** Representative western blots of 5-HTR1D protein in ZJW-treated and GR (5 μmol/L)-treated SW403 cells. **d** The corresponding semi-quantification data of (**c**). All values are shown as mean ± SD of three separate experiments, and significant values are indicated with asterisks (*P < 0.05, **P < 0.01 vs. control)

The choice of CRC model cells used in the present study is based on our initial screening for 5-HTR expression among various cell lines derived from digestive carcinoma. We found that 5-HTR1D is relatively highly expressed in SW403 cells. In our study, the data indicated that IC50 of ZJW extracts was 382.80 μg/ml, and IC10 was 117.98 μg/ml. The 3 different doses of ZJW extracts were less than IC10 of ZJW. Thus we use 100 μg/mL, 50 μg/mL and 25 μg/mL as high, middle and low concentration of ZJW in the experiments respectively. Thus, in current experiments, concentrations at 100, 50, and 25 μg/ml of the ZJW extracts were used, representing high (ZJW-H), medium (ZJW-M), and low (ZJW-L) doses, respectively. The results showed that, treatment of SW403 cells with 5-HTR1D antagonist GR127935 (GR) at 1, 5, 10 μmol/L down-regulated 5-HTR1D protein level in a dose-dependent manner (Fig. 1a and b). Likewise, compared to control, treatment of the cells with ZJW extracts also resulted in decreased 5-HTR1D expression in a dose-dependent manner (Fig. 1c and d).

### Results of cell viability assay in SW403 cells

**Fig. 2:**
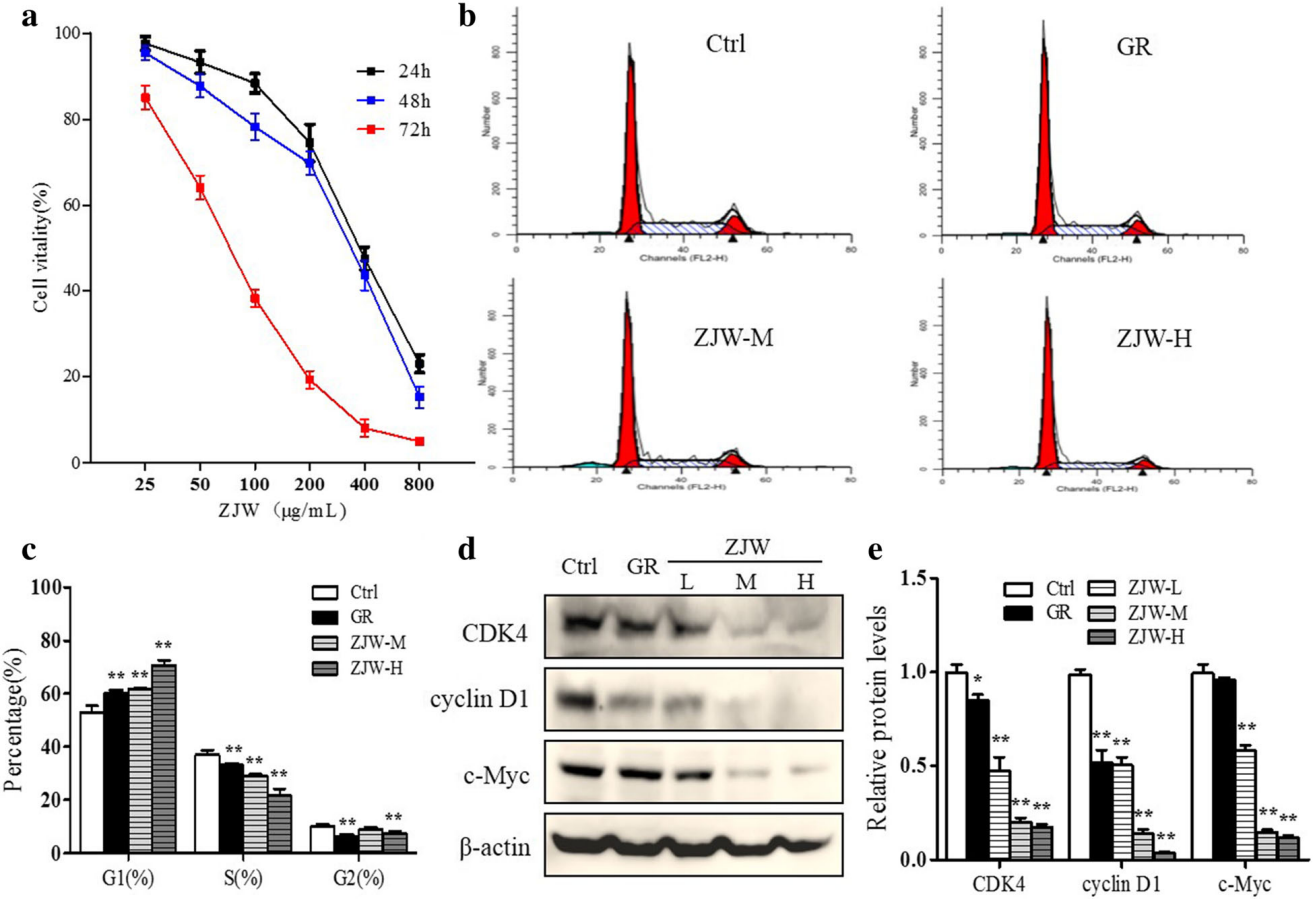
Effect of the ZJW extracts treatment on SW403 cell proliferation. **a** Cell proliferation was determined using the CCK8 assay. The total viability of cells treated with ZJW (25, 50, 100, 200, 400 and 800 μg/mL) for 24, 48 and 72 h were measured. **b** and **c** The percentage of cells at G1, S, G2 phase after treatment with GR (5 μmol/L), ZJW-M or ZJW-H for 24 h. **d** Representative western blots of CDK4, cyclin D1 and c-Myc in SW403 cells treated with GR (5 μmol/L), ZJW-L, ZJW-M or ZJW-H. **e** The corresponding semi-quantification data of **d**. All values are shown as mean ± SD of three separate experiments, and significant values are indicated with asterisks (*P < 0.05, **P < 0.01 vs. control)

The effect of ZJW on cell proliferation was determined by monitoring the cell viability using CCK8 assay. With increasing concentration of ZJW, SW403 cells demonstrated decreasing cell vitality in a dose-dependent manner (Fig. 2a). The effect of ZJW and GR on mitotic cycle distributions was subsequently investigated. Flow cytometry of SW403 cells showed that increased SW403 cells were arrested at G1 phase after 24 h treatment with medium or high doses of ZJW (Fig. 2b and c). The western blot results showed that the levels of cell cycle-related gene products, including cyclin D1, cyclin-dependent kinase 4 (CDK4) and c-Myc were attenuated with ZJW treatment as compared to those in control (Fig. 2d and e). The attenuation was most remarkable with ZJW-M or ZJW-H treatment.

### Results of apoptosis assay in SW403 cells

**Fig. 3:**
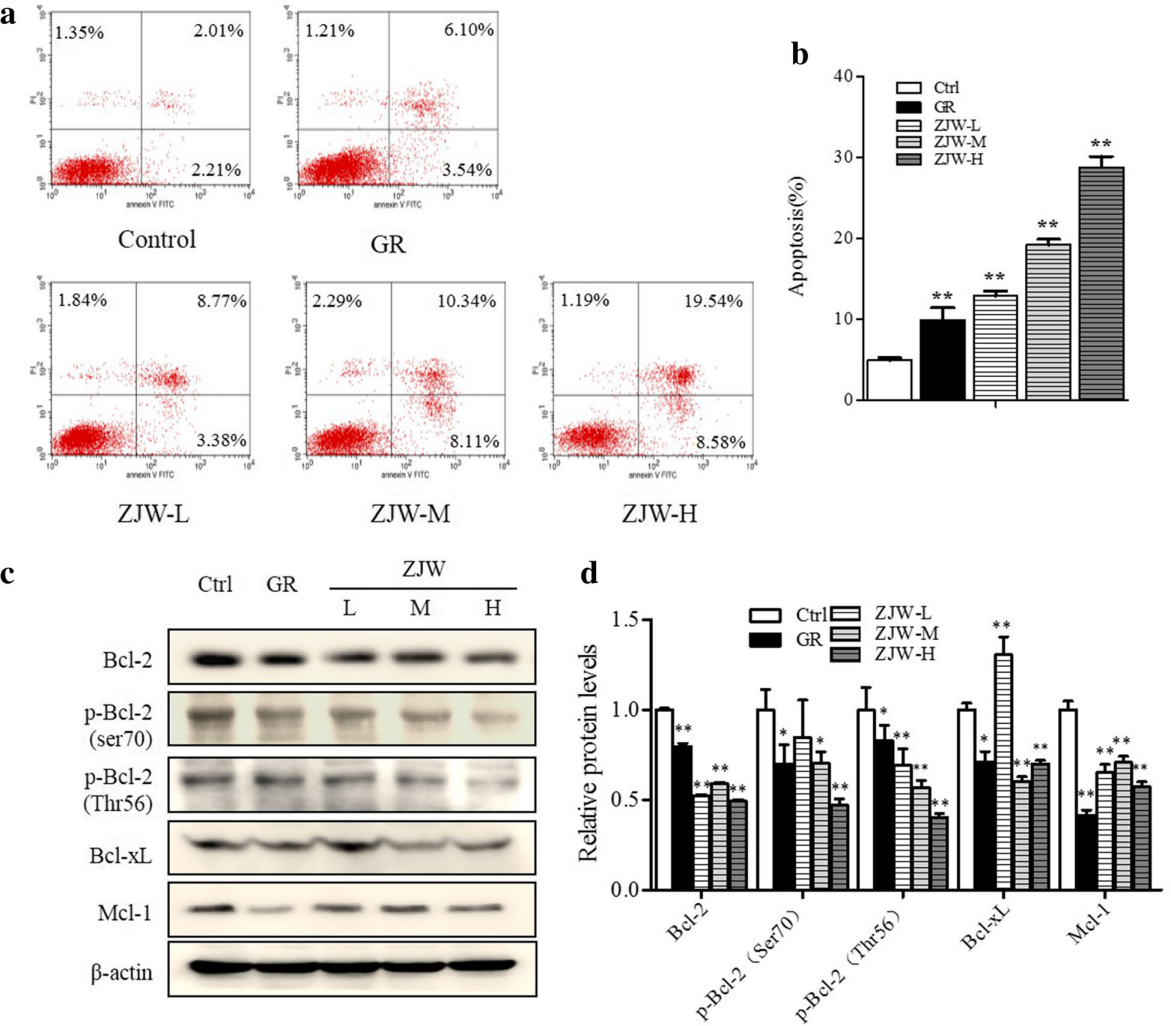
Effect of the ZJW extracts treatment on cell apoptosis. **a** Flow cytometric analysis of apoptosis of SW403 cell under ZJW-L, ZJW-M, ZJW-H, or GR (5 μmol/L) treatment conditions, compared to the control without any treatment for 24 h. **b** The percentage of cells underwent apoptosis. **c** Representative western blots of Bcl-2, p-Bcl2 (Ser70), p-Bcl-2 (Thr56), Bcl-xL and Mcl-1 in SW403 cells treated with GR (5 μmol/L), ZJW-L, ZJW-M or ZJW-H. **d** The corresponding semi-quantification data of (**c**). All values are shown as mean ± SD of three separate experiments, and significant values are indicated with asterisks (*P < 0.05, **P < 0.01 vs. control)

We next determined the effect of ZJW extracts on cell apoptosis. Flow cytometry demonstrated that, after treating with ZJW-M and ZJW-H for 24 h, the percentage of apoptotic cells was significantly higher in ZJW treated cells than that in control (Fig. 3a and b). The levels of B-cell lymphoma-2 (Bcl-2), p-Bcl-2 (Thr70), p-Bcl-2 (Ser56) were decreased significantly under ZJW-M and ZJW-H conditions (Fig. 3b). Expression of Mcl-1 and Bcl-xL was also down-regulated in cells treated with GR127935 or ZJW. The results showed that increased cell apoptosis upon ZJW treatment was closely associated with altered mitochondrial anti-apoptosis molecules.

### Results of cell migration and invasion in SW403 cells

**Fig. 4:**
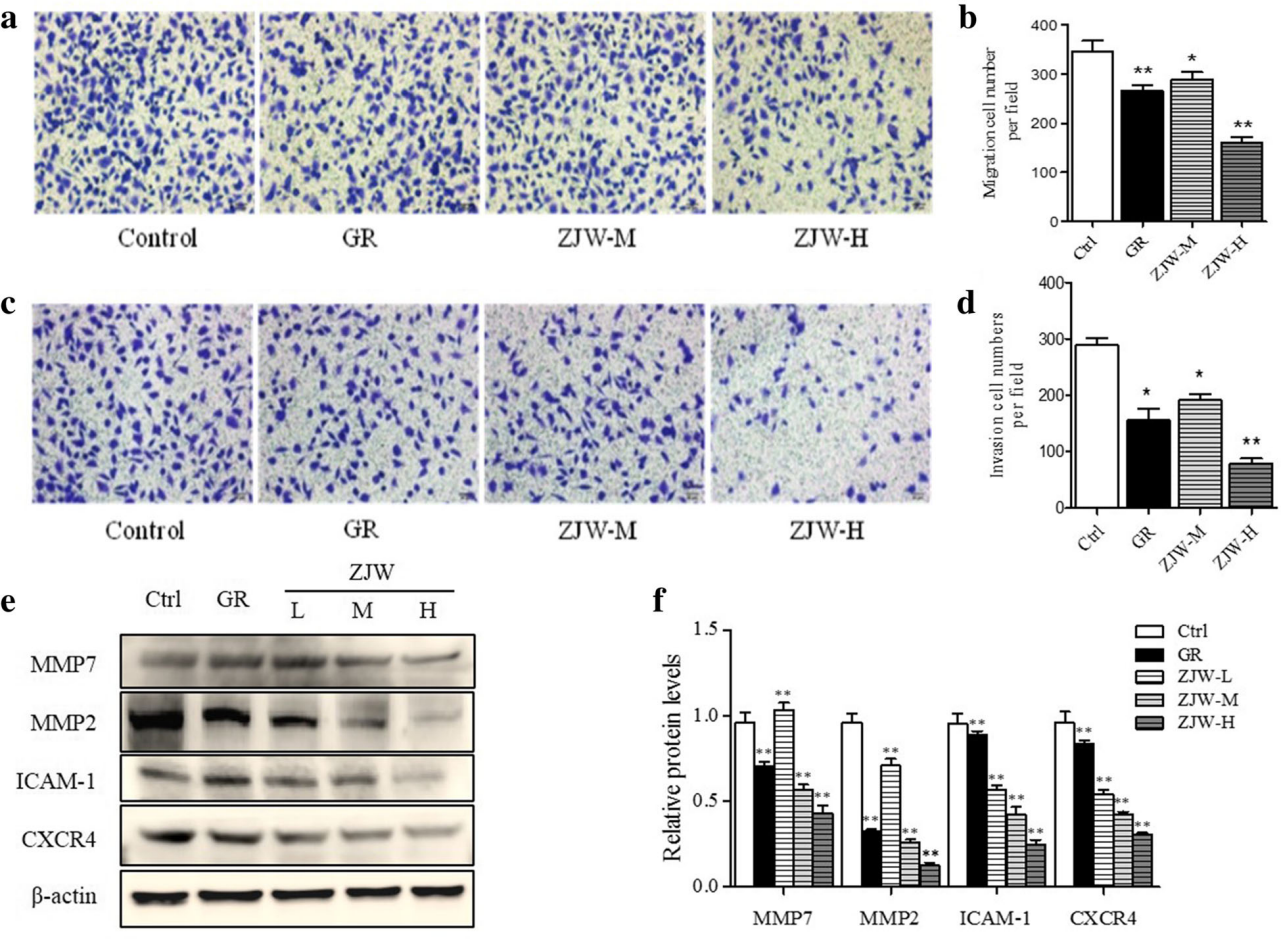
Effect of the ZJW extracts treatment on cell migration and invasion. **a** Migration assay of SW403 cells treated with ZJW-M, ZJW-H, GR (5 μmol/L) for 18 h. The underside of the filer was stained by crystal violet and observed through microscope (200×). **b** Migrated cells were counted under a microscope at a magnification of 200× (5 fields/filter). **c** Invasion assay of SW403 cells treated with ZJW-M, ZJW-H, GR (5 μmol/L) for 18 h. **d** Cells passed through the Matrigel and filer were counted under a microscope at a magnification of 200× (5 fields/filter). **e** Representative western blots of MMP2, MMP7, ICAM-1 and CXCR4 in SW403 cells treated with GR (5 μmol/L), ZJW-L, ZJW-M or ZJW-H. **f** The corresponding semi-quantification data of (**e**). All values are shown as mean ± SD of three separate experiments, and significant values are indicated with asterisks (*P < 0.05, **P < 0.01 vs. control)

The effect of ZJW extracts on SW403 cell migration and invasion was determined using the transwell assay. The number of cells that migrated through the chamber filter was significantly less in the group of GR or ZJW treatment than that in control group, and the effect was more profound when cells were treated with higher dose of ZJW (Fig. 4a and b). Similar results were obtained by the cell invasion assay (Fig. 4c and d). Thus, ZJW could inhibit the metastatic ability of colorectal cells.

To explore the mechanism further, we determined the level of matrix metalloproteinase 2 (MMP2), MMP7, intercellular adhesion molecule 1 (ICAM-1) and C-X-C chemokine receptor type 4 (CXCR4) that are related to metastasis. Western blot results showed that the levels of MMP2, MMP7, ICAM-1, and CXCR4 were all decreased in cells treated with ZJW in a dose-dependent manner (Fig. 4e and f).

### Wnt/β-catenin signaling expression after ZJW extracts treatment in SW403 cells

**Fig. 5:**
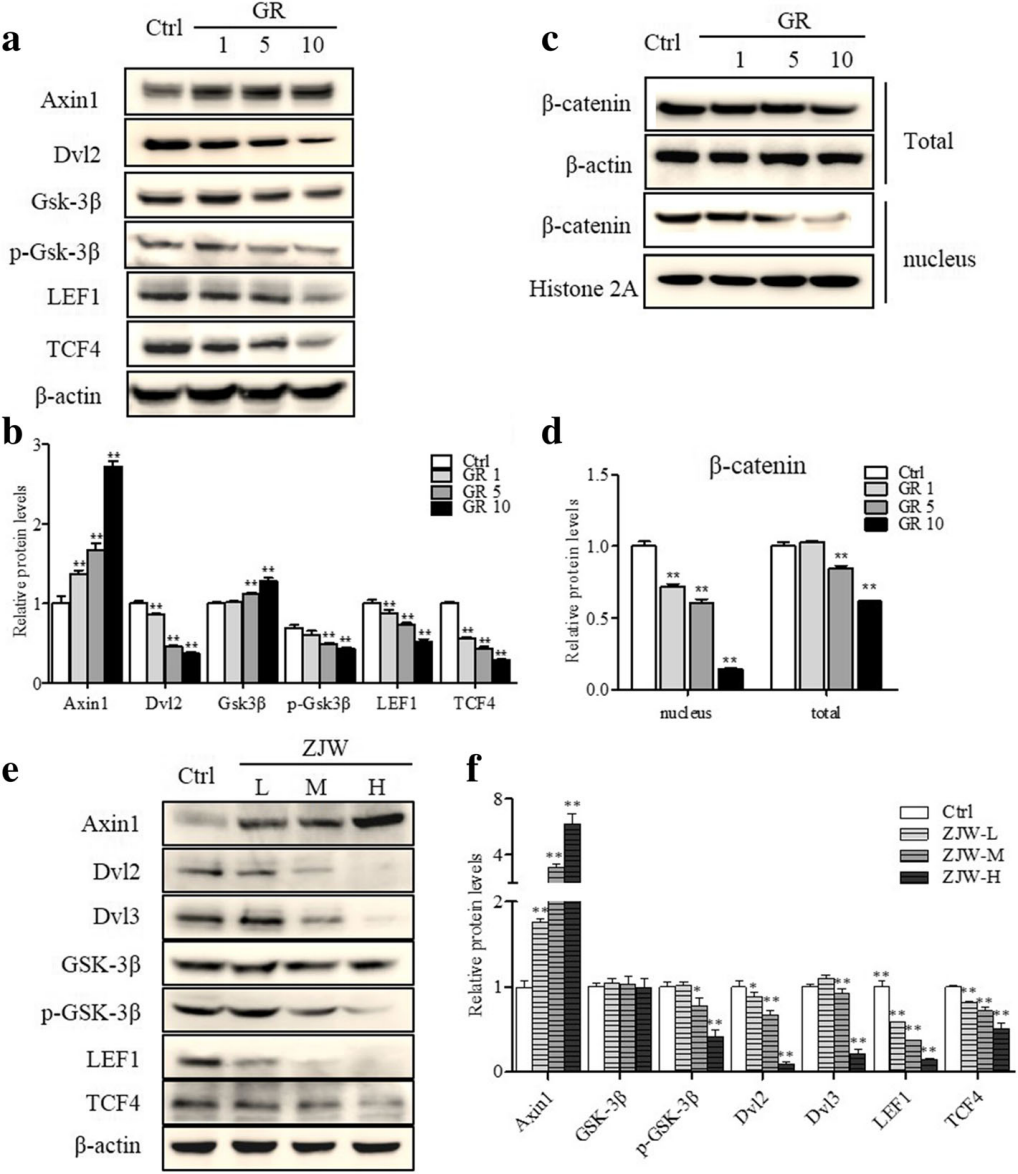
Effect of GR or ZJW treatment on the Wnt/β-catenin signaling pathway. **a** SW403 cells were treated with GR (1, 5, and 10 μmol/L, respectively) for 24 h. Representative western blots of Axin1, Dvl2, Dvl3, GSK-3β, LEF1, and TCF4 were shown. **b** Semi-quantification data of the Western blot relative to β-actin. **c** Western blotting of β-catenin in total cell lysate and nucleus in SW403 cells treated as (**a**). **d** The corresponding semi-quantification data of the Western blot relative to histone 2A. **e** SW403 cells were treated with ZJW-L, ZJW-M and ZJW-H for 24 h. Representative western blots for Axin1, Dvl2, Dvl3, GSK-3β, serine-phosphorylated-GSK-3β, LEF1 and TCF4 in SW403 cells were shown. **f** The semi-quantification data of the Western blot relative to β-actin. All values are shown as mean ± SD of three separate experiments, and significant values are indicated with asterisks (*P < 0.05, **P < 0.01 vs. control)

In order to define the underlying mechanism of the biological effects of ZJW on CRC cells, we further examined the Wnt/β-catenin signaling pathway because it was reported recently that the Wnt/β-catenin signaling is associated with the 5-HTR1D function [34]. As shown in Fig. 5a and b, SW403 cells treated with GR exhibited increasing expression, in a GR dose-dependent manner, of Axin1 and decreasing expression of Dvl2, p-GSK-3β, LEF1 and TCF4 as compared with control. Figure 5c and d showed, with GR treatment, β-catenin level significantly was decreased in nucleus. The total β-catenin level was decreased in cells cultured with 5 or 10 umol/L GR. Because nuclear accumulation of β-catenin is one of the key links of Wnt/β-catenin signaling transduction that promotes transcription of proliferation and metastasis related genes [35], these results suggest strongly that inhibition of 5-HTR1D can lead to suppression of Wnt/β-catenin signaling transduction.

The effect of ZJW treatment on increased Axin1 and concomitantly decreased Dvl2, Dvl3, LEF1 and TCF4 phenocopied the effect of GR treatment (Fig. 5e and f). In addition, although there was no change in the level of GSK-3β protein, ZJW treatment resulted in a significant reduction in serine-phosphorylated-GSK-3β (p-GSK-3β) (Fig. 5e and f). These results suggest that effect of ZJW treatment on the Wnt/β-catenin signal transduction resembles that of the 5-HTR1D antagonist GR127935.

## Discussion

Carcinogenesis is now considered as a result from a multitude of gene mutations [36], thus inhibition of a single gene product or cell signaling pathway is unlikely to prevent or treat cancer. The mechanisms vary considerably among various types of tumors. Alterations of different signaling pathways are involved in most of the pathological changes associated with these mechanisms. Many natural agents, in which TCM is included, have multi-targeting properties. We herein provide in vitro evidence that ZJW, a well-established TCM formula, has the potential for CRC intervention, which is accord to results obtained previously [6, 11]. It is a limitation not to perform cell vitality assay for ZJW in normal colorectal cells. However, the traditional Chinese herbal medicine ZJW has been used to treat gastrointestinal disorders for over 600 years in China. Nowadays, it is widely used in clinical medicine with proved security.

The present study showed that ZJW treatment, like that of 5-HTR1D antagonist, can attenuate proliferation of SW403 cells. The IC50 and IC10for SW403 cell of ZJW were 382.80 μg/mL and 117.98 μg/mLrespectively. Previous study had showed that the dosage of IC10 in HCT116/L-OHP cell was 50 μg/mL [6, 11]. This may be because of the resistance of different cell lines. Cells treated with ZJW exhibited increased G1 arrest, which was accompanied with decreased levels of CDK4, cyclin D1 and c-Myc. It is known that CDK4 and cyclin D1 are positively relative to cell mitosis, promoting G1/S transition. Thus, overexpression of CDK4 and cyclin D1 is invariably correlated with cancer progression. Expression of c-Myc is often associated with aggressive, poorly differentiated tumors, and is also required in transduction from G1 to S phase. Down-regulation of c-Myc gene expression is regarded as a therapeutic target for cancer treatment [37–40]. The increase in the proportion of cells present in G1 phase could be attributable to a c-Myc-dependent mechanism. The treatment with ZJW extracts also resulted in increased apoptosis in SW403 cells. Expression and activation of Bcl-2 family play an important role in the regulation of apoptosis. With the intervention of ZJW or GR, the level of the anti-apoptotic members of Bcl-2 family including Bcl-2, Bcl-xL and Mcl decreased remarkably. The activation of Bcl-2 was also attenuated dose-dependently by ZJW. In addition, cell migration and invasion were inhibited by ZJW extracts, which may be due to the significantly down-regulated expression of MMP2, MMP7, CXCR4 and ICAM-1.

Recent studies have shown that the level of 5-HTR1D expression was markedly increased in tumor as compared with normal colorectal tissue. Antagonist of 5-HTR1D, GR127935, could attenuate metastasis of CRC cell through regulating Axin1, the pivotal molecule in Wnt/β-catenin signaling pathway [34]. This suggests the relationship of 5-HTR1D and CRC. Interestingly, in the present study, we found ZJW treatment showed the same effect of inhibiting 5-HTR1D expression in CRC cells as GR did, which prompted us to propose and try to confirm that 5-HTR1D- Wnt/β-catenin maybe one of the underlying therapeutic mechanisms of ZJW for CRC. It is noteworthy that a drastic alteration in the level of 5-HTR1D, a trans-membrane G-protein coupled receptor (GPCR), occurs in cells that are treated with 5-HTR1D antagonist GR127935 or ZJW. Although it is well understood that conformational changes occur within the trans-membrane domains of GPCRs during the receptor activation/inactivation [41], relatively little is known about regulatory mechanisms controlling GPCR synthesis or turnover. To date, few report has shown that antagonist or agonist of 5-HTR1D alters the level of this GPCR expression. The decreased level of 5-HRT1D, in cells treated with the antagonist GR127935 or ZJW extracts, may be attributable to enhanced degradation in the lysosomes after receptor/antagonist endocytosis. How the interaction between 5-HTR1D and its antagonist affects the stability and/or the functionality of the receptor protein merits further investigation.

In addition to suppressing 5-HTR1D, ZJW treatment, as mentioned above, decreased the level of proteins involved in cell proliferation, apoptosis, and metastasis, which are all target genes of the Wnt/β-catenin pathway [35]. Measurement of several key components of the Wnt/β-catenin pathway, including Dvl, Axin1, P-GSK-3β, LEF1 and TCF4, provided further evidence suggesting that ZJW treatment attenuates cell proliferation through the Wnt/β-catenin signaling pathway. The current study has shown that Axin1 were up-regulated in cells treated with 5-HTR1D antagonist or ZJW, and the expression of Dvl2, Dvl3, P-GSK-3β, TCF4 and LEF1 were down-regulated, in an antagonist concentration dependent manner. Abnormal expression of these components is known to result in uncontrolled cell proliferation, loss of cell-cell adhesion, and increased cell migration [42]. Dvl may cause disintegration of Axin/APC/GSK-3β complex. Currently, the mechanism responsible for Axin1 upregulation is unknown, nor is it clear whether intermediary proteins are involved in signal transmission between Axin1 and Dvl. The cytoplasm β-catenin is phosphorylated by CK1 and GSK3β in the destruction complex, which could be recognized by ubiquitin E3 ligaseβ-TrCP and ubiquitylated for proteasome degradation. Prior studies have shown that Wnt signaling stimulates Akt, which in turn, in association with Dvl, enhances GSK-3β phosphorylation at Ser9, resulting in GSK-3β inactivation, causing increased β-catenin level [35]. In this study, although GSK-3β expression was not influenced by ZJW or GR, P-GSK-3β (ser9) level decreased remarkably, which means activated GSK-3β increased. Thus, ZJW or GR treatment may lead to decreased level of β-catenin and low expression of wnt target genes such as cyclin-D1, c-Myc, MMP2 and MMP7. The nuclear β-catenin was shown reduced by GR in our results, while the regulation of cytoplasmic β-catenin level as well as the effect of ZJW on β-catenin need proved in our future work. Previous studies have demonstrated that downregulation of the Wnt/β-catenin induces apoptosis in a variety of human cancer cell, suggesting that the Wnt/β-catenin pathway may be associated with cellular apoptosis. Therefore, ZJW induced apoptotic effect in CRC cell by regulating Bcl-2 proteins, possibly through Wnt/β-catenin pathway.

**Fig. 6:**
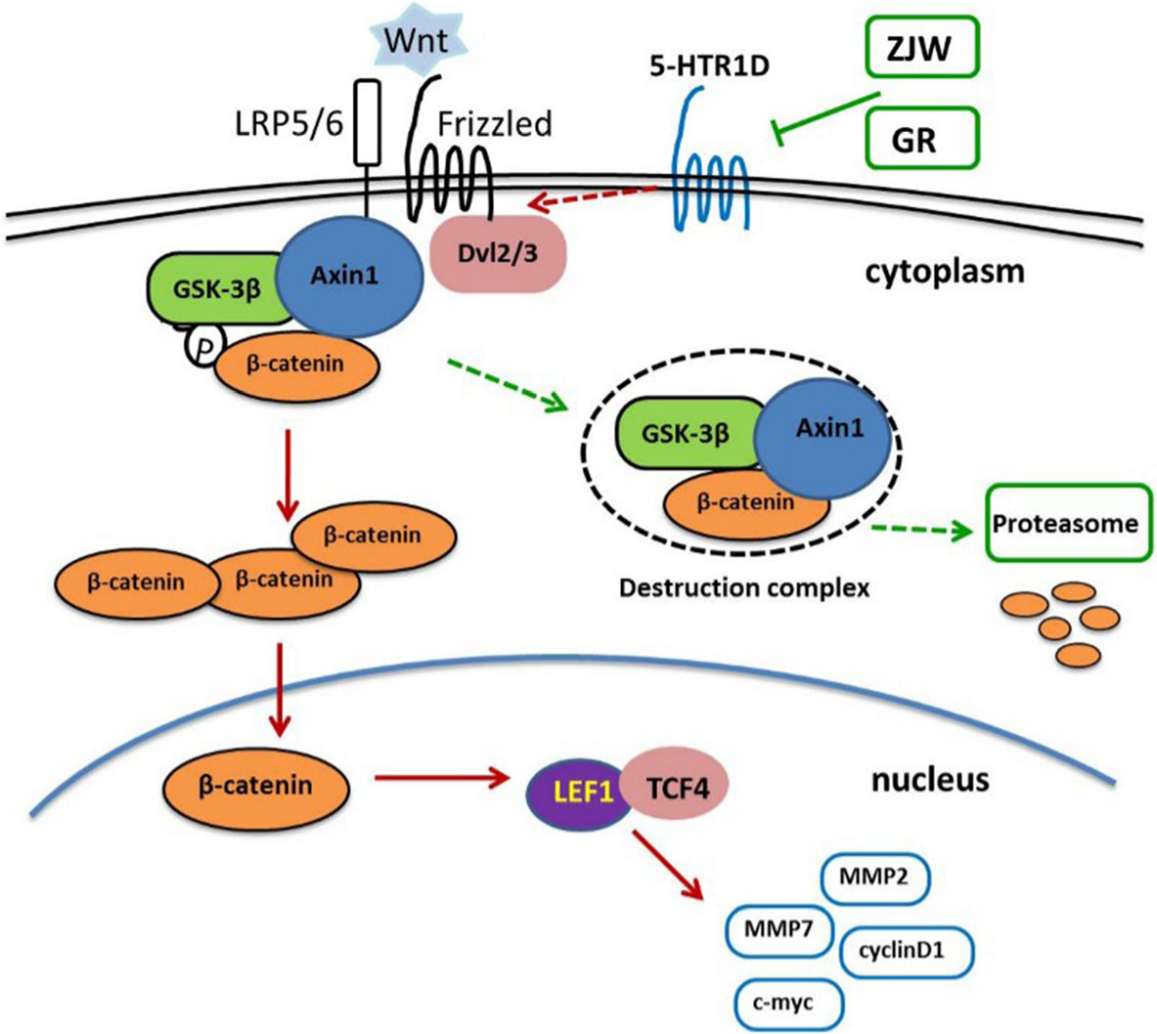
A schematic model for ZJW extracts in inhibiting 5-HTR1D and attenuating Wnt/β-catenin signaling pathway. Wnt/β-catenin signaling is activated by the binding of Wnt ligand to Frizzled receptor and LRP5/6 co-receptors, leading to the recruitment of Dvl and destruction complex to the membrane, which inactivates destruction complex, causing accumulated β-catenin to enter nucleus and activate target gene transcription (red arrows). 5-HTR1D may play a role in promoting the Wnt/β-catenin signaling at upper stream, leading to stabilization of β-catenin (red arrows). The effect of ZJW extracts on Wnt/β-catenin resembles that of GR127935 (GR), an antagonist of 5-HTR1D, in inhibiting (green arrows) the Wnt/β-catenin signaling through enhanced β-catenin degradation, thus prohibiting the signaling transduction

The current study has also shown that the transduction of Wnt/β-catenin pathway appears to be sensitive to the level of 5-HTR1D expression. Our data have indicated that 5-HTR1D might play a role in influencing the Wnt/β-catenin signaling at upper stream, leading to stabilization of β-catenin in cells. It is rather remarkable that ZJW treatment helps activate the β-catenin destruction complex, leading to degradation of β-catenin, possibly through down-regulation of 5-HTR1D. Expressions of the Wnt/β-catenin target genes, e.g., TCF4 and LEF1, were decreased by the ZJW treatment. The demonstrated effect of ZJW treatment on the expression of key components of the Wnt/β-catenin pathway and its target gene products is summarized schematically in a model depicted in Fig. 6. This model postulates that ZJW treatment attenuates CRC cell survival and metastasis through regulation of the 5-HTR1D-Axin1-TCF4/LEF1 axis of the Wnt/β-catenin signal transduction. The model also postulates that downregulation of the Wnt/β-catenin signaling might regulated by elevated Axin1, which results in increased degradation of β-catenin. However, the possibility that ZJW might exert its anti-cancer effect by directly targeting at some downstream protein factors remains to be determined.

In conclusion, the present studies thus provide experimental evidence that in the CRC cell line SW403, the level of 5-HTR1D expression can be effectively suppressed by the ZJW extracts treatment, which in turn leads to attenuated cell growth and cell invasion. The antagonizing effect of the ZJW extract in suppressing 5-HTR1D expression in SW403 cells is almost indistinguishable from that of the authentic 5-HTR1D antagonist GR127935. In addition, treatment with the ZJW extracts can achieve the same inhibitory effect on the canonical Wnt signaling pathway through suppressing the β-catenin target gene expression. Our study thus provided a possible mechanistic explanation for the potential therapeutic effectiveness of ZJW extracts in treating CRC. However, more direct evidences are required to confirm the link of ZJW-5-HTR1D and Wnt/β-catenin pathway.

## Conclusions

Downregulation of 5-HTR1D expression by the ZJW extracts treatment results in suppression of CRC cell growth and invasion, which is associated with inactivated Wnt/β-catenin signaling.

## Abbreviations

APC: Adenomatous polyposis coli
Bcl-2: B-cell lymphoma-2
CDK4: Cyclin-dependent kinase 4
CFDA: China Food and Drug Administration
CRC: Colorectal cancer
Dvl: Dishevelled
EC: Enterochromaffin cells
GSK3β: Glycogen synthase kinase 3β
GR: GR127935
HPLC: High-performance liquid chromatography
MMP2: Matrix metalloproteinase 2
SSRI: Selective serotonin-reuptake inhibitors
TCF/LEF: T cell factor/lymphoid enhancer factor
TCM: Traditional Chinese medicine
ZJW-L: Low dose of ZJW
ZJW-M: Medium dose of ZJW
ZJW-H: High dose of ZJW
ZJW: Zuo Jin Wan
5-HTR: 5-hydroxytryptamine receptor
5-HT: 5-hydroxytryptamine
